# Artificial Intelligence in Social Health: A Narrative Review of Uses, Advantages, Challenges, and Future Directions

**DOI:** 10.3390/healthcare14142114

**Published:** 2026-07-14

**Authors:** Yousif M. Elmosaad

**Affiliations:** Department of Public Health, College of Applied Medical Sciences, King Faisal University, Al Ahsa 31982, Saudi Arabia; yelmosaad@kfu.edu.sa

**Keywords:** artificial intelligence, social health, AI technology, digitalization, social inequalities, AI algorithm

## Abstract

Artificial intelligence (AI) is deeply integrated into daily life. Emerging evidence suggests AI may help change the dynamics of social relationships by influencing social interactions, connectivity, and interpersonal relationships, and by providing new avenues for communication and contributing to improved social well-being. Therefore, this review aims to explore the potential of artificial intelligence (AI) technologies as a tool to enhance social health, focusing on current applications, advantages, challenges, and ethical considerations associated with their implementation, as well as opportunities for future development. The literature on the relationship between the connectedness of social health dimensions and AI as a tool to better understand how interactions with AI technologies may influence social well-being. In this current review, key terms such as “Artificial Intelligence”, “Social Health”, “social inequalities”, “AI algorithm”, “AI technology”, “social connection”, “digital communication”, “social participation”, “social support”, “social isolation”, “loneliness”, “mental wellbeing”, were used to search relevant literature on Google Scholar, PubMed, Scopus and Web of Sciences. In addition, relevant aspects of the multidimensional impacts of AI on social health dimensions are also discussed. The use of AI technologies by individuals within societies was found to hold profound potential to reshape social health through enhancing social relationships, bridging communication gaps in diverse populations, stimulating social dynamics, and understanding human emotions. It may contribute to reducing social inequalities, promoting equity, accommodating individual differences, and enhancing the effectiveness of many tasks in the social and health care systems through deep learning, natural language processing, and machine learning techniques. This reduces social exclusion and increases accessibility and quality of health and social services. However, AI has also posed distinguishable challenges to its adoption, specifically in terms of data quality, privacy and security, algorithmic bias, ethical issues, public trust and acceptance, and regulatory and policy gaps. Evidence suggests that building public trust in the future of AI in social health requires interdisciplinary collaboration among health providers and professionals, social scientists, community members, and policymakers. Such collaboration is crucial to ensure that AI platforms do not perpetuate social inequalities or biases by maintaining transparency, explainability, and demonstrated effectiveness. In conclusion, the integration of AI into social health dimensions holds promise for social health transformation. As we move forward, several key areas need to be addressed to develop a robust governance and regulatory framework, along with ethical guidelines to ensure privacy protection, respect for human rights, transparency, and the promotion of the common good.

## 1. Background

The constitution drafted by the World Health Organization (WHO) in 1946 emphasizes the importance of addressing the challenges of treatment and medical care. It also highlights the need to tackle the social roots of health problems. Moreover, its founders considered health to be “a state of complete physical, mental, and social well-being and not merely the absence of disease or infirmity” [1,2,3]. This definition presents a holistic view, underlining the importance of social, physical, and mental aspects, and also explores dimensions of complete well-being and the absence of disease or infirmity [4,5,6,7]. Although this definition reflects that social health is a cornerstone of the well-being of both individuals and the community as a whole, the well-being of the individuals is interrelated with the quality of living, interactions with other people, adherence to societal values, customs, morals, and responses to the social institutions [8,9,10]. These elements highlight an acknowledgment of the crucial role of social connections in health.

Around three decades after the adoption of the WHO constitution, it was observed that different meanings of social health began to appear in research across disciplines [8]. A growing body of literature indicates that agreement on the definition of social health was impossible at the time, although there was consensus on social factors influencing individual health and on their key components of overall well-being for the individual and the community [3,10,11,12].

However, many attempts across different scientific disciplines, such as sociology and epidemiology, and in line with the WHO constitution, support investigations to construct a definition of social health based on its components: loneliness, belonging, and social integration [13,14,15]. Based on this context, Waite in 2018 defined social health as “Adequate and well-functioning social relationships, adequate social support, little or no social strain, some social participation, social inclusion in one’s society, strong and well-functioning social networks, and, perhaps, sexuality as one desire” [16,17]. Moreover, after a careful review of related studies conducted by other researchers in the field of social health and well-being, Doyle and Link defined social health as “the adequate quantity and quality of relationships in a particular context to meet an individual’s need for meaningful human connection” [10]. Thus, we observe that these definitions differ from one another, but they share some essential and common characteristics, among which is the individual’s need for adequate social relationships and meaningful human connections.

For the purpose of this review, social health is defined as “an individual’s and community’s ability to establish and maintain meaningful social relationships, access social support, participate in social and community activities, and experience social inclusion, connectedness, and sense of belonging”. This definition provides a clear framework for evaluating how emerging technologies, particularly Artificial Intelligence (AI), influence social health outcomes.

Based on this framework, AI applications were categorized according to whether they employ a direct or indirect influence on social health. Direct applications include AI technologies that enhance social interaction, strengthen social support networks, reduce loneliness and social isolation, facilitate communication, promote community participation, and improve social inclusion. In contrast, indirect applications include AI systems primarily designed to improve health care delivery, diagnosis, administrative efficiency, resource allocation, surveillance, and population health management. Although these applications may contribute to social health by improving access to health care and service effectiveness, their impact on social health is mediated through broader health system improvements rather than through direct enhancement of social relationships and community engagement.

Given the rapidly evolving nature of AI technologies, the evidence base regarding their impact on social health varies considerably across application areas. In this review, a distinction is made between outcomes supported by current evidence, emerging findings requiring future validation, theoretically plausible mechanisms derived from established social and behavioral theories, and future opportunities that remain largely speculative. This distinction is intended to provide a balanced interpretation of the literature that helps contextualize the strength of existing evidence and avoid overstating the demonstrated effects of AI on social health outcomes.

Concerning the concept of social health, which has emerged recently and plays a significant role in individuals’ overall health, balance, and wellbeing, also plays an extensive role in health outcomes [15,18]. Evidence increasingly suggests that individuals with stronger social connections tend to have longer lives than those with fewer connections. This may be due in part to reduced exposure to health-risky behavior such as obesity, smoking, and alcohol abuse, which are correlated with an increased risk of chronic disease [19,20,21].

Given the importance of social health, evidence shows that high-performing health systems worldwide incorporate social health indicators and metrics into their health strategies, aiming to enhance overall population health status [22]. This approach is supported by several studies that emphasize the importance of social interaction and human relationships as fundamental factors in improving the quality of life for individuals and societies [23,24,25].

Based on this evidence, it is clear that social health represents a multidimensional concept whose components interact to shape the mental health and physical well-being of individuals and communities. Moreover, these findings highlight the necessity of extending scientific understanding of the relative and integrative impact of social health on overall health status and adopting an advanced integrative approach grounded in the latest theoretical and technological developments. Such an approach should take into account the interconnection among the multiple dimensions of social health as a fundamental pillar for improving the quality of life.

Regarding technology use and its impacts on social health at the individual level, based on insights from the previous studies, it was shown that technology changes the dynamics of social relationships by influencing the ways of an individual’s interaction and facilitating social engagement and enhancing interpersonal relationships by providing new opportunities for communication with people [26,27]. In the case of using technology as a tool in social health, digital platforms, mobile applications, wearable devices, telemedicine, online support networks, and online services can be used to monitor and manage health outcomes. In some situations, these tools can reduce face-to-face interaction and privacy concerns; however, they help overcome barriers to social interaction by improving access to health care, promoting health literacy, and providing accessible, affordable communication platforms that foster social interaction in various forms, including people with disabilities [28,29,30].

As these technologies, such as AI, increasingly penetrate human lives, AI is defined as “technology that enables computers and machines to simulate human learning, comprehension, problem solving, decision making, creativity and autonomy”, demonstrating its capability as an innovative technology able to perform tasks that typically require human intelligence such as reasoning, learning, perception and understanding [31,32]. AI platforms can manage large amounts of data, recognize patterns, make predictions, and develop actionable knowledge based on information. These range from simple rule-based programs to advanced Machine Learning (ML), Deep Learning (DL), and Natural Language Processing (NLP), which can improve their performance over time and are widely used in applications [33,34]. These advanced applications have enabled the development of ambient intelligence, which is increasingly being applied in health care settings. Ambient intelligence involves the use of contactless sensors and contact-based wearable devices to collect and interpret data, and to assess the quality and nature of care delivered across the entire health care system [35,36].

Accordingly, the recent literature in the basic health sciences has investigated the challenges of implementing AI in resource-constrained settings. However, the implications for social health have not yet been thoroughly examined. Rapid and transformative developments in AI have outpaced our understanding of its potential impact, especially in the area of social health, where it may significantly affect health outcomes [37]. This knowledge gap highlights the importance of exploring the impact of AI on social health dimensions as well as the associated regulatory and governance frameworks. This narrative review examines how AI technologies could improve social health for individuals and communities, focusing on current applications, opportunities for future development, implementation challenges, and the ethical considerations associated with their use. The review also aims to provide a comprehensive understanding of how AI applications contribute to supporting social health interventions. Furthermore, the review seeks to fill the existing knowledge gaps concerning the application of AI technologies in social health by providing a comprehensive overview of their benefits, challenges of AI applications, and future development prospects. The review also culminates in a series of recommendations aimed at strengthening the practices of individuals, communities, and healthcare systems in promoting social health. Therefore, the aim of this narrative review is to explore the potential of artificial intelligence (AI) technologies as tools to enhance social health, with a focus on current applications, advantages, challenges, ethical considerations, and opportunities for future development.

## 2. Methods

This study employed a narrative review approach to critically examine the impact of Artificial Intelligence (AI) on Social Health, with particular focus on current uses, advantages, challenges, and future opportunities, associated risks, and ethical considerations. The review aimed to provide a conceptual and thematic synthesis of the existing literature rather than a quantitative aggregation of findings.

### 2.1. Conceptual Scope and Definition

To ensure conceptual clarity, this review defines social health as “An individual’s ability to establish and maintain meaningful social relationships, participate in social activities, access social support, and experience social inclusion, connectedness, and community belonging”. Key dimensions include social connectedness, communication, social support, loneliness, participation, and psychological well-being. The review differentiates between two categories of AI applications. The first includes AI applications that directly impact social health outcomes such as social connectedness, loneliness reduction, communication, participation, inclusion, community engagement, social support, and psychological well-being. The second includes AI applications primarily designed for administrative or system performance, including health care delivery, diagnosis, surveillance, and workflow optimization. These include only when their indirect relevance to social health is explicitly demonstrated. This distinction is applied consistently throughout the review to ensure conceptual focus and coherence.

### 2.2. Search Strategy

A comprehensive literature review was conducted using electronic databases: Google Scholar, PubMed, Scopus, and Web of Science. The search was performed from January to the fourth week of March 2026 and included studies published between January 2000 and March 2026. Search terms were developed to capture literature related to AI and social health. The primary search string combined terms related to the AI and social health, including “artificial intelligence” OR “AI”, OR “machine learning”, and “social health” OR “social connection” OR “social isolation” OR “loneliness” OR “social support” OR “digital communication” OR “social participation”, OR “mental wellbeing”.

### 2.3. Eligibility Criteria

Studies were included if they were published in peer-reviewed journals and written in English, and examined AI applications with direct relevance to social health outcomes. Eligible studies addressed dimensions of social health such as social connectedness, communication, social participation, loneliness reduction, interpersonal interaction, and psychological well-being. Empirical studies, theoretical papers, conceptual analysis, and review articles were included.

Studies were excluded if they focused primarily on hospital administration, healthcare efficiency, disease surveillance without direct implications for social health, or AI-assisted clinical diagnosis and treatment planning. Studies focused purely on technical algorithm development without directly addressing social health outcomes were also excluded. Furthermore, conference abstracts and articles without an accessible full text were not considered for inclusion.

### 2.4. Study Selection

The titles and abstracts were initially screened for relevance. Potentially eligible articles were reviewed afterward for full text. The Duplicate records identified across the database were removed before the full-text assessment. Studies that met the inclusion criteria were critically reviewed and categorized according to the thematic areas, including AI application for social connectedness, AI-supported communication, psychological well-being, ethical and privacy concerns, and risks related to dependency, exclusion, and social issues.

The initial search yielded 186 full-text articles. After screening and eligibility assessment, 98 articles met the inclusion criteria and were included in the final review.

### 2.5. Data Extraction and Thematic Synthesis

The relevant information was extracted from each included study, including publication characteristics, study design, AI technology platforms, target population, social health dimensions examined, and key findings, the selected literature was critically reviewed and categorized in tor thematic dimensions including AI application for improving social connectedness and reducing loneliness, AI supported communication, social interaction, AI enabled psychological support, ethical, privacy and governance considerations, risk related to dependency, exclusion, algorithmic bias and social inequalities. The evidence was synthesized using a thematic narrative approach to identify recurring patterns, emerging trends, opportunities, and challenges regarding the role of AI -related social health outcomes.

### 2.6. Conceptual Framework

To ensure conceptual coherence, the findings were interpreted and discussed through the use of established theories relevant to social health, including Compensation Theory, Social Cognitive Theory, Social Influence Theory, and Social Identity Theory. These theories guided the organization and interpretation of evidence by explaining the mechanisms through which AI technologies may influence social connectedness, social participation, social support, and broader social health outcomes. The theoretical frameworks were used throughout the review to structure the discussion and to distinguish social impacts associated with the use of AI technologies.

## 3. Theoretical Framework

This review aims to explore the impacts of artificial intelligence (AI) on social health; it employs a theoretical framework that includes relevant theories and concepts significant for examining this phenomenon. This framework enables an in-depth, systematic exploration of how the connectedness dimension of social health interacts with AI as a tool that influences social health outcomes. The most important approach in this situation is social influence theory, which provides a structured framework for examining the mechanisms and conditions under which social pressure shapes how individuals adjust their attitudes, behavior, and social interactions to meet the demands of a social environment [38,39]. Based on this background, specifically, we can utilize the Social Influence Theory to explain the use of AI technologies as communication mediator platforms providing information, personalized feedback, recommendations, and social cues, making it a powerful agent for changing the dynamics of social relationships by influencing the ways of individuals’ interaction, decision making, and facilitating social engagement and enhancing interpersonal relationships. Moreover, it enables a deeper understanding of both overt compliance and deeper internalization processes that drive social health [40,41]. Conversely, the theory in the context of AI can be applied to explore how virtual representations and interactions, such as users’ self-esteem, emotional states, particularly through comments on social media, can contribute to stress, depression, and anxiety, highlighting the importance of considering social influence in the use of AI [42,43].

In addition, compensation theory offers insight into how the use of AI applications helps overcome social limitations by providing valuable resources that support coping with stress and anxiety, thereby further enhancing their mental and social well-being [39]. In the context of AI and social interaction, this principle has been used to design systems that detect and respond to users’ needs. For example, virtual assistance can identify signs of loneliness and social stress through behavioral cues and provide support to compensate for these gaps, such as engagement in social activities. Moreover, AI Platforms can predict compensatory online behavior, such as increased use of social media use, participation in virtual communities, and adoption of interaction strategies to promote social inclusion and well-being. By integrating Compensation Theory, AI technologies can enhance social interaction, creating a more adaptive, supportive social environment that addresses problems associated with social health [42,43,44].

Furthermore, Social Identity Theory (SIT) posits that individuals drive part of their self-concept from their membership in social groups, with these group affiliations influencing their attitudes, behaviors, and social interactions [45]. SIT also examines processes such as social categorization, identification, and comparison, which contribute to group differentiation. These mechanisms help explain how group membership shapes conformity and cooperation, which can be quantified through indicators of group identification and associated behavioral outcomes [46]. In relation to AI, SIT helps to investigate how AI systems shape group dynamics, social interactions, and cohesion. In this way, the previous study reported that digital interactions, driven by AI platforms, can significantly shape self-esteem and social relationships by influencing how an individual interacts and by reinforcing identity-based communities [47,48].

Cognitive-behavioral theory offers another framework for understanding how engagement with technologies can affect social health. Social Cognitive Theory (SCT) illustrates that behavior change is shaped by the interplay of personal, behavioral, and environmental factors [49]. This theory also highlights several key determinants of behavior; for instance, social outcome expectations reflect an individual’s beliefs about how other people will evaluate them for performing a given behavior and how others value the outcomes of that behavior [50]. With regard to the use of AI platforms, the theory can enhance self-efficacy through mastery experiences, social modeling, emotional states, and social persuasion [51].

Collectively, these theories provide a conceptual framework for understanding how AI influences social health through multiple pathways. Throughout this review, they are used to interpret potential benefits and risks of AI applications. Particularly, they help explain how AI may promote social participation, strengthen social support networks, and foster social inclusion. Though also contributing to unintended consequences such as social exclusion, distorted social interaction, and technological dependence. The following sections apply these theoretical concepts to examine current applications, challenges, and future directions of AI in social health.

**Figure 1:**
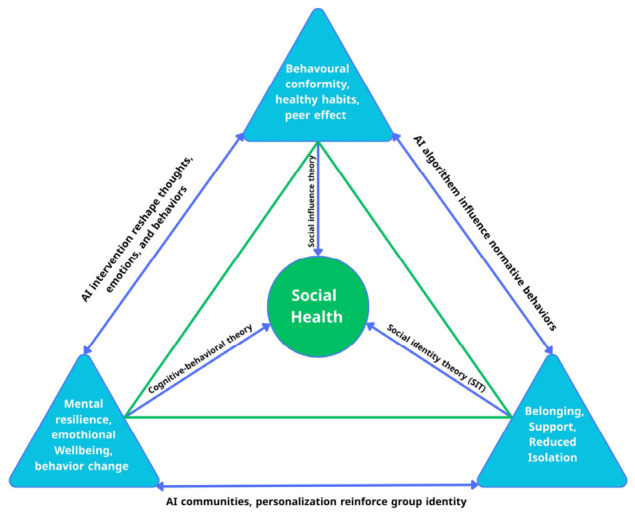
Theoretical framework for the application of AI.

In summary, based on the key insight highlighted in the theoretical frameworks mentioned above (Figure 1), this study will investigate the multidimensional impacts of AI on social health dimensions.

## 4. Social Health and Artificial Intelligence (AI)

According to the WHO, the overall global health status has improved; nevertheless, many social dimensions still need further improvement [52]. To improve these areas, health systems have integrated advanced technologies, particularly artificial intelligence (AI), which can have a significant social impact on healthcare. This impact occurs through mechanisms that directly influence social interactions and engagement, rather than through improvements in health care systems. Direct mechanisms include a strong social network and health education programs that foster social skills and health literacy. These factors enhance social coherence, connectedness, reduce isolation, and improve resilience, all of which play an extensive role in social health outcomes [53,54,55]. Therefore, the digitalization of health care services, combined with the integration of social health dimensions as key factors that shape human health, allows users to better understand social circumstances and foster the development of new forms of social networking, thereby strengthening social relationships [56].

AI may influence social health through both direct and indirect pathways. Direct AI applications, such as asocial robots for companionship, online peer support platforms, community engagement chatbots, and mental health conversational agents, are specifically designed to address core dimensions of social health. These technologies enhance interaction, strengthen social support networks, may help reduce loneliness and social isolation, facilitate communication, promote community participation, and improve social inclusion by strengthening social connectedness and engagement, and developing a sense of belonging. In contrast, AI applications designed primarily to improve health care system performance, including predictive analytics for vulnerable populations, administration automation, diagnostics decision support, resource allocation systems, and population health management tools, affect social health indirectly. Although these technologies are not intended to strengthen social relationships directly, they may improve physical and mental health outcomes, increase access to health care, reduce healthcare costs, support informed decision-making, and decrease reliance on social services. Through these mechanisms, they create conditions that facilitate social participation, inclusion, and engagement in social life [57,58,59].

AI models in health care systems are being used to influence social dimensions of health. One of their most prominent applications is the ability to integrate and analyze factors connected with the social dimensions of health, such as the social environment, which shapes individuals’ health outcomes [60]. In this context, previous studies have reported that AI applications serve as tools that help health care professionals extract social determinants of health data from medical records. This enables the identification of vulnerable social groups, as well as broader social norms, beliefs, and regulatory influences, thereby guiding health care professionals in identifying, designing, targeting, and modifying interventions more efficiently [61,62]. Moreover, AI applications such as automated triage systems, virtual assistants, and telemedicine platforms, when used appropriately, can reduce health inequities through improving access to care [63]. In this sense, we can say that AI strengthens the association between healthcare systems and the social environments.

Machine learning is used in AI-powered community platforms and community-level AI application models to promote social interaction. By improving communication and decision-making, fostering inclusion and interaction, and increasing the efficacy and efficiency of community services, they make social systems more responsive [64,65]. From this viewpoint, AI serves as an assisting tool that aids communities in preserving their social connections and general health.

AI applications at the individual level are increasingly used in the domains of social health, including social interaction, economic, environmental, and structural determinants, by providing accessible and affordable communication platforms that reduce disparities, improve social service delivery, and foster interaction in various forms [18,29,66]. Among artificial intelligence (AI) methods, Natural Language Processing (NLP) and Machine Learning (ML) are the most widely used techniques to identify, extract, and analyze health information related to the social determinants of health [67,68]. However, recent studies highlight that combining social determinants of health with other health indicators enhances the development of risk stratification models, which can more precisely identify and predict individuals and communities at higher social risk [69,70]. In addition, studies have shown that Artificial Intelligence embedded in social media and communication platforms, expressively alters the way people interact, communicate, build connections online and offline, and shapes what persons see and engage with their families, friends, and colleagues worldwide, thereby shaping broader social structures and cultural patterns and diversity of social contact [39,71]. Furthermore, engagement in digital communication through AI applications is one of the most prominent ways to influence social relationships, with AI integrated into social networking tools such as chatbots, virtual assistants, and AI-driven messaging tools. Algorithms organize content, recommend friends, and enhance social connectivity by supporting individuals in maintaining their relationships across distances and discovering new communities. Also, offering immediate responses and emotional support [72]. According to the recent body of research, AI provides social support by shaping social relationships through its influence on behavioral patterns and the perception of social norms, as well as by reinforcing certain habits, which indirectly guide users’ social behaviors and interactions [73,74].

In summary, the previous discussion demonstrates that the applications of AI across health care systems, communities, and individuals have a significant social impact. At the health systems level, AI facilitates the transformation of administrative procedures and supports the effort to mitigate social determinants of health. In the community, AI applications such as social robots for companionship, online peer support platforms, community engagement chatbots, and mental health conversational agents directly enhance social health by strengthening social connectedness, facilitating participation and interaction, and fostering collective social commitment support. While at the individual level these technologies expressively alter the way people interact, communicate, and build social connections in online and offline contexts, collectively these technologies can play a significant role in promoting social connectedness, reducing social isolation, and promoting overall well-being.

AI has made remarkable progress in understanding, rapidly analyzing large and complicated datasets, and generating actionable recommendations that aid in the process of decision-making and enhance the efficiency of numerous tasks in healthcare systems. For instance, AI in augmented care enhances clinical decision-making, supports personalized treatment, and improves workflow efficacy. These advancements have attracted the attention of health care professionals and policymakers at both national and international levels, driving investments in AI technology [37,75]. These innovations can benefit health care transformation and reduce social inequalities in access to care [76]. Through AI empowered telehealth platforms and virtual assistants, underserved communities can access medical care, mental health, social health, and health promotion services [77]. Furthermore, the body of research reported that a prominent advantage of AI platform applications in healthcare is that they lead to better treatment outcomes, improved disease diagnosis, large-scale data analysis, improved workflows, health communication, reduced workload and costs, and improved overall efficiency in healthcare systems [78,79,80]. Additionally, the literature from around the world shows that integrating AI into health care enhances disease prevention, emergency response, and public health surveillance by enabling early detection of diseases and outbreaks through analysis of large datasets, such as electronic health records and social media data. This helps reduce the social burden of disease and protect societies [81,82].

Regarding the advantages of AI in social health, it offers several benefits. It is particularly valuable for supporting mental health, as the technology can interpret emotional cues from a variety of data sources [83]. The work of Habermann and a study by Glaz et al. in 2021 [84] explain how the combination of natural language processing (NLP) and sentiment analysis enables algorithms to read and understand human emotions expressed in text. NLP models have also been used to monitor mental health self-disclosure on social media, specifically Twitter, facilitating the detection of depression, anxiety, abuse, and loneliness, and suicidal thoughts, and enabling the assessment of suicide risk. These models are used to provide a complete understanding of emotions and psychological well-being, specifically engaging elderly, vulnerable, and isolated individuals [84,85,86,87]. From the perspective of Compensation Theory, AI -mediated communication tools, virtual companions, and digital support platforms many compensate for deficits in social interaction and emotional support by providing accessible forms of communication, assistance, and monitoring, thereby helping to reduce social isolation and loneliness in certain populations and settings [42,43,44].

A recent literature review identified AI as supporting social contact by bridging communication gaps in diverse populations, which encourages communities to adopt healthier habits and improve collective social wellness. AI further enhances social interactions by creating personalized content and activities that adapt to the users’ evolving needs, preferences, behaviors, and abilities. Its advanced capabilities facilitate expressive communication and comprehension across a broad range of topics, extending beyond simple question-and-answer exchanges [84,88]. These capabilities can be understood through the SCT, which proposes that personalized feedback, adaptive guidance, and interactive technologies enhance self-efficacy, support informed decision-making, and encourage social engagement [50,51]. By facilitating communication and providing tailored support. AI may empower individuals to participate more actively in social and community life.

AI also stimulates social engagement by taking different perspectives and incorporating features such as politeness and playfulness, enhancing users’ social experiences. Its personalization capabilities allow it to account for individual differences in age, culture, and cognitive ability, particularly when supported by memory functions that leverage past interactions, which may foster stronger feelings of social connectedness and belonging. Furthermore, AI can be integrated across a variety of platforms, including virtual and augmented reality, digital games, social robots, and smartphone applications [89]. These capabilities can be integrated through Social Identity Theory, which suggests that AI-embedded communities may strengthen social participation, social inclusion, reinforce group membership, and promote a sense of belonging among different populations. By facilitating shared experiences and integration among individuals with the similar need, identities, or interests [45].

Furthermore, AI can influence social perceptions, attitudes, and behavior through personalized recommendations, social feedback mechanisms, and digital interactions [89]. According to SIT, these features may encourage adaptation of healthier behaviors, facilitate community participation, and shape social perceptions within digital environments [45]. The flexibility of AI enables its integration across a variety of platforms, including virtual and augmented reality systems, digital games, social robots, and smartphone applications [89]. Collectively, these abilities place AI as uniquely capable of supporting, enhancing, and strengthening social interaction, social engagement, and community connectedness.

Table 1 highlights the multidimensional contribution of AI to social health at individual, community, and system levels. The findings indicate that AI platforms enhance service efficiency, facilitate personalized and preventive interventions, strengthen social connectedness, and improve access to social support. At the individual level, AI enhances diagnostic accuracy and service efficiency, supports early detection of psychological vulnerabilities, provides personalized recommendations, improves access to continuous, stigma-free health care adaptive assistance, and facilitates informed decision-making through data-driven insight. At the community level, AI may contribute to social inclusion, strengthen social networks, and enhance community engagement. It also improves the efficiency and effectiveness of social services delivery, facilitates the identification of vulnerable populations requiring targeted interventions, and supports the planning, coordination, monitoring, and evaluation of community programs. At the systems level, AI optimizes administrative processes, enhances operational efficiency, reduces health care costs, strengthens workforce capacity, and supports evidence-based policymaking. It further improves the responsiveness of health and social care systems by facilitating resource allocation and strategic planning. Additionally, AI analyzes social health indicators and population characteristics to inform target policies, preventive strategies, and community interventions. Collectively, this emerging evidence suggests AI may support a transformative approach for strengthening social health through integrated, data-driven, and population-centered strategies.

## 5. Challenges in Integrating AI in Social Health

**Figure 2:**
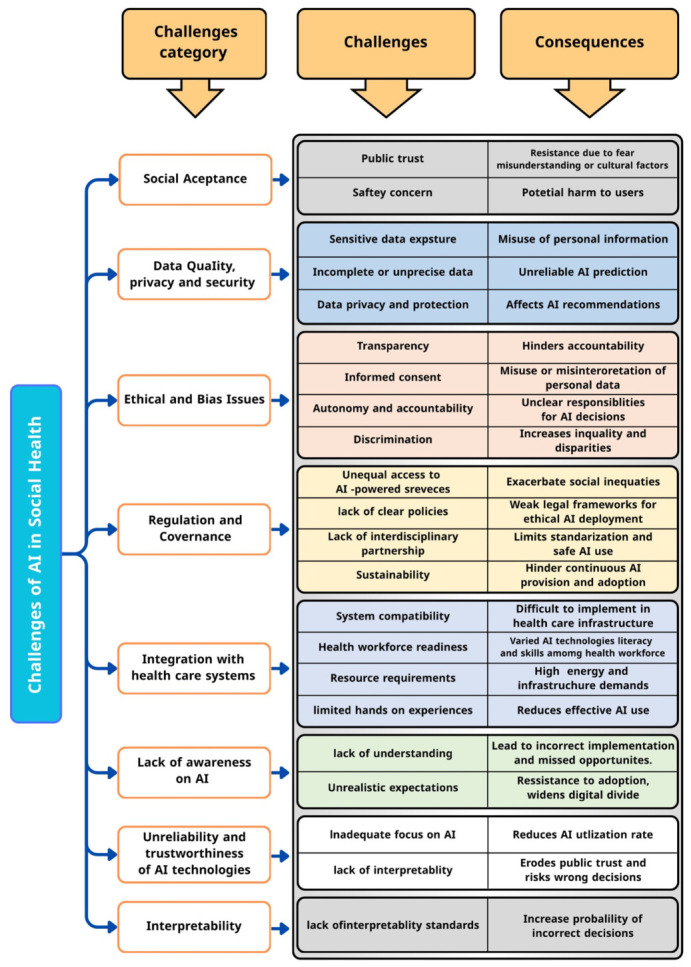
Summarized AI challenges in social health.

Figure 2 presents a conceptual synthesis of the major challenges associated with the application of AI in social health. While AI holds significant potential to enhance social connectedness, improve access to social support, and support early identification of social and psychological needs, its implementation is constrained by a range of interrelated challenges across individual, community, and system levels.

At the individual level, these include concerns regarding privacy and data security, reduced human interaction, overreliance on AI platforms, misinformation, and potential impacts on autonomy and trust. At the community level, challenges involve digital exclusion, unequal access to technology, algorithmic bias, and the potential reinforcement of existing social inequalities, particularly among vulnerable populations.

At the system level, key issues relate to governance, transparency, accountability, ethical oversight, data quality, regulatory compliance, and the interpretability of AI-driven decisions.

Collectively, these challenges highlight that the integration of AI into social health requires a balanced, ethically grounded approach that ensures technological innovation is aligned with the principles of equity, inclusivity, and responsible governance.

Failure to address these challenges may undermine public trust, widen social inequalities, compromise decision quality, and limit the effectiveness, safety, and sustainability of AI-driven social health interventions. These consequences underscore the need for robust governance frameworks, transparent and ethical AI practices, improved digital literacy, and effective integration strategies to ensure the safe, equitable, and sustainable use of AI in social health.

Despite AI having the potential to transform social health data, it can support monitoring the directions of the risk factors by analyzing the sociodemographic characteristics data, behavioral data, and environmental data. AI can also assess the impact of interventions planned to change adverse social health factors [37]. While it has also posed distinct challenges to its adoption [90], specifically in terms of technical, ethical, and social concerns, lack of awareness, quality of data, and professional liability have been illuminated as key challenges [91,92]. Ethical equity, accountability, and data privacy are among the primary social challenges that require strengthening rules, regulations, and laws, as well as robust digital infrastructure and workforce capacity development [93,94].

The application of AI in health services and its social dimensions through social media platforms provides a broad range of challenges, including maintaining the privacy of individuals’ confidentiality and data security; preventing the potential misuse of personal information; respecting the ethical limits of technology innovation; and assessing the actual effect of AI technology on social groups [95]. It also causes significant risks due to the widespread dissemination of low-quality information. An AI model may raise an issue of discrimination, especially among marginalized populations, as AI can be trained on data from specific demographic groups, which may introduce bias into datasets [96].

Additionally, a study highlighted that inaccurate data could lead to discriminatory outcomes in terms of resource allocation; residents in disadvantaged communities may be systematically excluded by AI models [91]. While AI has the potential to improve social trust in this technological decision-making process [97]. The AI algorithmic systems, along with the absence of transparency and interpretability of AI algorithms, and the biases embedded in algorithms, remain major challenges. These factors generate uncertainty and resistance, amplify societal inequalities, and exacerbate existing health disparities, thereby hindering the effective implementation of AI within the social system [98,99].

The challenges associated with AI integration can also be understood through the theoretical framework adopted in this review. From the perspective of Social Influence Theory, AI may shape behavior, attitudes, and social norms in ways that increase dependence on algorithmic recommendations and facilitate surveillance and behavioral monitoring [40,41,42,43]. Compensation Theory suggests that although AI-mediated communication and support systems can supplement social interactions, excessive reliance on these technologies may reduce opportunities for meaningful human relationships and community engagement [42,43,44]. SIT highlights the risk that biased algorithms and unequal access to digital technologies may reinforce stigmatization, social exclusion, and existing social inequalities, particularly among vulnerable populations [45]. Similarly, SCT suggests that limited transparency, reduced interpretability, and overreliance on automated decision-making systems may undermine individual autonomy, self-efficacy, and critical judgment. Together, these theoretical perspectives demonstrate that the social health consequences [50,51] of AI depend not only on technological capabilities but also on the social settings in which these technologies are designed, developed, and governed.

Although integrating AI requires technological infrastructure and experience, which can pose challenges in resource-limited settings [100], one of the most significant challenges is the lack of a comprehensive framework for assessing AI integration across complex social dimensions. Moreover, the limited availability of high-quality data for training and evaluating AI models can result in inaccurate predictions and algorithmic biases [80].

With regard to rules and policy challenges, despite the increasing use of AI technologies across different areas, many legal frameworks remain under development or are inconsistent, leading to uncertainty regarding accountability, data protection, and ethical oversight [101]. Existing regulations fail to provide clear instructions on the matter of managing a large volume of sensitive personal data, including data security, algorithm validation, and liability in case of AI-driven errors, probably compromising social rights, safety, and disproportionately affecting marginalized communities and resulting in discriminatory outcomes across different social and legal contexts [102].

Emerging governance frameworks and technical solutions aim to mitigate AI-associated risks. These include algorithmic auditing, bias detection tools, fairness-aware machine learning techniques, stakeholder engagement, and participatory design approaches to ensure responsiveness to marginalized and vulnerable populations, thereby reducing discriminatory outcomes [103,104]. Additionally, ongoing international initiatives such as the European Commission’s AI Act emphasize accountability, transparency, and human-centric design as key requirements for trustworthy AI deployment [105]. Integrating these regulatory, technical, and participatory approaches, along with interdisciplinary collaboration and ethical safeguards, enables the responsible implementation of AI in a social context.

Addressing these challenges requires raising public awareness of AI technologies, fostering interdisciplinary partnerships, and developing a strategic plan dedicated to enhancing awareness, health workforce training, scientific studies, and sustainable development to support effective AI applications to social issues. Recently, there has been a growing global effort to address these concerns, including increased funding for applied research about the social and ethical consequences of AI and the proliferation of initiatives aimed at promoting responsible technology. These efforts include the establishment of organizations and guiding principles designed to support ethical AI development and governance, such as the cooperation on AI, OpenAI, the Foundation for Responsible Technology, Governance and the Ethics of Artificial Intelligence Initiative, and the Principles for Accountable Algorithms [98,102]. All these initiatives collectively seek to promote transparency, accountability, and socially centered values in the growing use of AI and their translation into practice.

## 6. Future Direction for AI Use in Social Health

The future of AI offers significant potential for creating advanced tools for enhanced social health, particularly through the development of ethical and transparent AI models that foster social interaction, accountability, inclusivity, and public trust [71,100]. In this context, recent studies and a growing body of research focus on the development of population-explainable AI systems to enhance transparency, trust, and accountability, while preventing the perpetuation of social inequalities and understanding of social determinants of health [103,104].

Future AI initiatives in social health should also be guided by the theoretical perspectives adopted in the review to minimize potential harms. Social Influence Theory emphasizes the importance of designing AI systems that encourage healthy behavior, community participation, and social engagement without undermining individual autonomy and users’ ability to make independent choices [42,43]. Compensation Theory supports the development of AI tools that complement rather than reduce individuals’ relationships and existing social support networks, ensuring that technology enhances meaningful social connections [42,43,44]. SCT underscores the value of transparent, user—centered technology that enhances self-efficacy, digital literacy, and informed decision making [51]. Similarly, SIT highlights the need for inclusive and equitable AI systems that strengthen the social sense of belonging, promote social inclusion, and reduce disparities across different population groups [47,48]. Integrating this theoretical principle into AI system design, implementation, and governance may help mitigate risks associated with technological dependence, social exclusion, and distorted social relationships and interactions.

Recently, there is evidence suggesting that one of the key areas of AI is the improvement of personalized health and preventive care by integrating data from wearables and lifestyle habits [105]. This approach can lead to proactive social and healthcare systems where the health outcomes are predicted and managed before they spread. Similarly, AI can significantly address health disparities by optimizing health systems, cutting costs, improving allocation of resources, promoting social interaction, and enabling the precise acquisition and dissemination of health information to tackle social determinants of health [81].

AI technologies are becoming increasingly prevalent in our daily lives. In this context, the development of a robust governance framework, regulations, and ethical guidelines can create significant opportunities, such as enabling human self-realization by supporting society members in pursuing their own characteristics, interests, skills, and cultural, intellectual, and other dimensions. Moreover, AI can enhance human agency, without removing human responsibility, improving the possibilities for social intervention and supporting societal cohesion, collaboration, and shared moral systems [98]. Simultaneously, artificial intelligence will provide opportunities to enhance and improve the capabilities of both individuals and society by managing, may contribute to disease prevention through early identification of risk factors, and support for preventive intervention and disease treatment.

AI is particularly valuable from a behavioral health perspective, where data derived from mobile apps and social media platforms can be analyzed to monitor health behaviors, such as physical activity, diet, and social connectedness. Moreover, these data can be used to evaluate the impact of interventions designed to change these behaviors in the short and long term [106,107]. Additionally, AI algorithms can be used to extract people’s feelings, views, opinions, beliefs, personality, and attitudes from content shared on social media platforms and in online interactions [108]. This approach has been applied in the environmental health field to monitor air quality and in mental health applications [109].

AI is expected to play significant role in social health, communication targeting specific population groups with precise health messages culturally appropriate in multiple languages and across level of literacy and responsive to the community needs and detecting health misinformation circulates online and engaging communities in real time for example the World Health Organization (WHO) implemented AI based chatbots on messaging platforms such as WhatsApp to convey timely health information during COVID-19 pandemic and also capable to provide immediate responses to people’s inquires, helping to detect misinformation, guiding individuals to toward reliable health information sources thereby strengthening communication and community engagement [110,111].

Other promising areas of emerging AI applications are personalized health care, smart assistive technologies, and AI-driven decision support, which contribute to social health by automating routine community interactions and addressing long-term implications, including ethical considerations and the integration of AI into daily life. These applications efficiently process and summarize large amounts of health information, such as policies, regulations, guidelines, health messages, and scientific reports. Such capabilities can significantly empower individuals in their health-related decision-making processes, enhancing their quality of life [112,113].

Recently, the growing reliance on data-driven decision-making across the health sector has raised concerns about data privacy. This indicates that the future use of AI in social health requires a robust regulatory framework to ensure that appropriate ethical safeguards are upheld, particularly to protect privacy and the security of health data. These safeguards should align with the guidance and standards set out by the National Institute of Standards and Technology (NIST), considering technical challenges such as secure data storage, encryption methods, and anonymization techniques, which are among the most effective data processing and access control mechanisms [114,115].

Such a framework promotes equity to prevent health disparities, enhances community engagement in the design, deployment, and assessment of AI tools to ensure that tools are contextually relevant and trusted by the communities, and encourages innovation [30]. The WHO has set out principles for designing such frameworks that focus on respecting human rights, ensuring transparency, and promoting the common good. These principles are built on recent research that employed a variety of methods and tools and also support technological progress and innovation [116,117]. Regarding the future Need for Standardization and Governance in social health, it is essential to effectively guide the deployment of technologies such as AI to manage the scale and speed of social health interventions [106].

The adoption of a standardized governance framework is necessary to provide a structured mechanism to enable the implementation of trustworthy AI, ensuring that ethics, fairness, transparency, accountability, and socio-technical factors are fully considered throughout the AI implementation process. That framework could help to reduce risks, including safety hazards and discriminatory outputs [118,119,120].

By establishing oversight mechanisms that address bias, equity, transparency, ethics, data confidentiality, and safety, social institutions can ensure that all factors influencing trust are appropriately regulated, thereby supporting the safe and trustworthy implementation of AI models, for example, privacy and federated learning, which could enable the use of sensitive social health data without compromising privacy [121,122].

Moreover, given the complex nature of social dimensions, additional governance domains, structures, and processes may require providing appropriate oversight and accountability. This includes tracking bias in each domain, ensuring accountability, and implementing standardized mechanisms across all stages of AI development, application, evaluation, and long-term use in a sustainable manner. This comprehensive governance ensures that AI models and platforms not only provide technical support but also focus on diverse and marginalized populations in data collection and raise awareness to promote trust and unbiased outcomes. Besides investments in capacity building and technical literacy, which will help societies harness the advantages of AI in a safe and sustainable manner.

**Figure 3:**
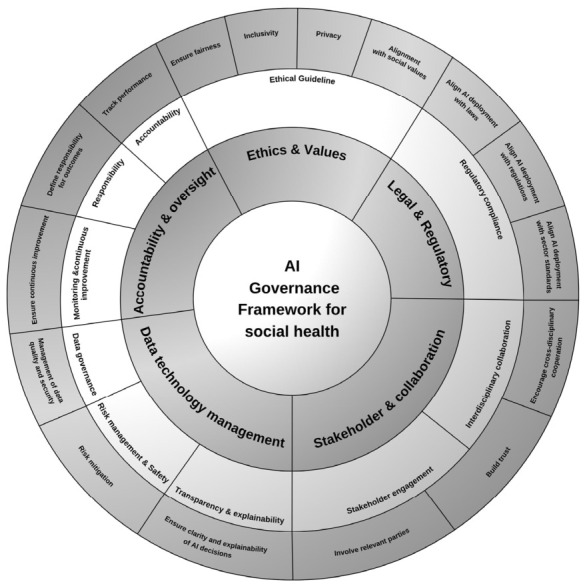
Proposed standardized AI governance framework for social health.

In summary, Figure 3 presents an author-developed conceptual framework for AI governance in social health, synthesized from established principles in AI ethics, digital health governance, and responsible innovation literature. The framework integrates four interrelated dimensions: ethical and values, legal and regulatory compliance, accountability and oversight, and data and technology management, supported by stakeholder collaboration.

The ethics and values dimension emphasizes foundational principles such as privacy, inclusivity, fairness, transparency, and alignment with the social values in the deployment of AI systems. The legal and regulatory dimension ensures compliance with relevant laws, policies, and sector-specific standards governing data protection, algorithmic use, and digital health applications. The accountability and oversight dimension focuses on responsibility, monitoring, continuous evaluation, and governance mechanisms that ensure safe, reliable AI performance in social health contexts.

The data and technology management dimensions address data governance, system security, risk management, and the transparency and explainability of AI-driven decisions. The stakeholder and interpersonal collaboration dimension ensures the involvement of relevant actors, including policymakers, healthcare professionals, technologists, and community representatives, to enhance trust, contextual relevance, and shared decision-making.

Overall, these dimensions form an integrated governance structure aimed at ensuring that AI systems in social health are ethically grounded, legally compliant, technically robust, and socially responsive.

## 7. Conclusions and Recommendations

The incorporation of AI into social dimensions is not just influencing social well-being and quality of life by providing powerful tools for social interaction, facilitating online social connections, analyzing large, comprehensive datasets, and generating actionable recommendations that support the process of decision-making. AI may contribute to reducing social inequalities, accommodating individual differences in culture, age, and mental ability, and enhancing the effectiveness of many tasks in the health systems. Together, these capabilities position AI as a promising tool for supporting, enhancing, and strengthening social connectedness. In addition, AI links individual behaviors, system interactions, and well-being outcomes, enabling increasingly personalized approaches that account for individual health and the social decision-making process. However, although AI demonstrates considerable potential to support social health, the strength of evidence varies across applications and outcomes. Current evidence most strongly supports AI’s role in enhancing service accessibility, facilitating communication, and providing personalized support in selected contexts. The emerging evidence regarding long-term effects on social connectedness, social inclusion, loneliness, community participation, and community health outcomes remains limited and continues to evolve. Consequently, many anticipated advantages should be viewed as promising possibilities rather than established outcomes, highlighting the need for rigorous longitudinal and real evaluation studies. At the same time, the integration of AI raises critical ethical, governance, and social challenges. Addressing these issues needs reasonable AI development, robust data governance frameworks, transdisciplinary research, and forward-looking policies that promote equitable, transparent, and trustworthy social and health outcomes. The future of AI in social health extends beyond predictive accuracy and mechanization; it requires interdisciplinary partnership, a comprehensive ethical and policy framework, inclusive approaches, privacy protection, user-centered technologies, and a standardized governance structure. When these elements are integrated thoughtfully and responsibly, AI has the potential to reduce health disparities, promote equity, and enhance the responsiveness of social systems. Furthermore, AI continues to transform health care and social health through deep learning, natural language processing, and machine learning techniques. As these technologies evolve, their successful implementation will depend on balancing innovation with ethical oversight, transparency, social inclusion, and accountability to ensure that AI contributes positively to social health.

## 8. Limitations of the Review

Despite the significance of the insights provided in this review, several limitations should be acknowledged. As a narrative review, this review may have a higher potential for selection and interpretation bias, lower reproducibility, and reduced reliability compared with a systematic review, due to the absence of a standardized methodology for study selection, data extraction, and analysis. Therefore, future systematic reviews and meta-analyses are recommended to provide stronger evidence regarding the role of artificial intelligence in social health.

## Figures and Tables

**Table 1:** Summary of the advantages of Artificial Intelligence in social health.

Advantage	Individual Level Effects	Community Level Effects	System Level Effects	References
Health Systems Strengthening and Capacity Building	Enhances diagnostic accuracy. efficiency, and individual health outcomes.	Strengthens health workforce capacity and supports evidence-based decision- making in community health programs, identifying vulnerable social groups and mitigating health inequalities.	Optimizes administrative processes, reduces health care costs, strengthens health workforce capacity, and supports health system governance.	[18,41,46]
Promotion of Social connectedness and engagement	Reduce isolation, facilitate communication, and promote inclusion	Strengthens social networks, enhances community cohesion, and improves community engagement.	Support the design of socially responsive programs and community-based interventions.	[18,47,52,54,55,60,61,62]
Personalized and Adaptive Social Health Interventions	Delivers tailored recommendations and support based on individuals’ preferences and needs.	Enables targeted interventions for at-risk groups and populations with specific needs.	Enhances the responsiveness and effectiveness of health and social care programs.	[75,76]
Enhanced Access to Social Support and Community Resources	Provides continuous, accessible, and stigma-free access to health care and social support resources regardless of cost, location, or time constraints.	Strengthening support networks promotes equitable community participation.	Facilitates efficient resource allocation and services accessibility.	[32,59]
Equity-Focused Support for Vulnerable and Marginalized Populations	Identifies individuals at risk, provides timely assistance and support.	Promotes inclusion, participation, and engagement of marginalized groups.	Supports equity-oriented resource allocation and informs social and healthcare policies.	[57,58,72,73,74]
Early Identification of Social and Psychological Risks	Detects early indicators of stress, loneliness, and social withdrawal.	Identifies emerging social needs and population-level risk trends.	Supports preventive strategies and community-level intervention planning	[60,61,62,72,73,74]
Optimization of Social and Health Services Delivery	Reduces administrative burden and improves service accessibility and user experience.	Enhances coordination and operational workflow across community programs.	Automates routine processes, improves operational performance, and streamlines service delivery.	[30,63,66,67,68]
Data-Informed Planning and Decision Making	Supports informed decision-making through personalized insights and predictive analysis	Enhances community program planning, monitoring, and evaluation	Analyzes social health indicators to guide policy development, strategic planning, and population-level interventions	[30,63]

Logical flow: advantages of AI at the individual, community, and systems level.

## Data Availability

No new data were created or analyzed in this study. Data sharing is not applicable to this article.
